# High individual variability in the transcriptomic response of Mediterranean mussels to *Vibrio* reveals the involvement of myticins in tissue injury

**DOI:** 10.1038/s41598-019-39870-3

**Published:** 2019-03-05

**Authors:** Magalí Rey-Campos, Rebeca Moreira, Valentina Valenzuela-Muñoz, Cristian Gallardo-Escárate, Beatriz Novoa, Antonio Figueras

**Affiliations:** 1 Institute of Marine Research (IIM), National Research Council (CSIC), Eduardo Cabello, 6, 36208 Vigo, Spain; 2 Laboratory of Biotechnology and Aquatic Genomics, Interdisciplinary Center for Aquaculture Research (INCAR), University of Concepción, P.O. Box 160-C, Concepción, Chile

## Abstract

Mediterranean mussels (*Mytilus galloprovincialis*) are sessile filter feeders that live in close contact with numerous marine microorganisms. As all invertebrates, they lack an adaptive immune response and how these animals are able to respond to a bacterial infection and discriminate it from their normal microbiome is difficult to understand. In this work, we conducted Illumina sequencing of the transcriptome of individual mussels before and after being infected with *Vibrio splendidus*. The control mussels were injected with filtered seawater. We demonstrate that a great variability exists among individual transcriptomes and that each animal showed an exclusive repertoire of genes not shared with other individuals. The regulated genes in both the control and infected mussels were also analyzed and, unexpectedly, the sampling before the injection was considered a stress stimulus strong enough to trigger and modulate the response in hemocytes, promoting cell migration and proliferation. We found a clear response against the injection of filtered seawater, suggesting a reaction against a tissue injury in which the myticins, the most expressed antimicrobial peptides in mussel, appeared significantly up regulated. Functional experiments with flow cytometry confirmed the transcriptomic results since a significant alteration of hemocyte structures and a decrease in the number of hemocytes positive for myticin C were found only after a *Vibrio* infection and not observed when mussels were bled before, generating a tissue injury. Therefore, we report the involvement of myticins in the response to a danger signal such as a simple injection in the adductor muscle.

## Introduction

In the past years, there has been a considerable effort to understand the molecular basis of many biological processes of non-model organisms, such as bivalves. Although they are cultured worldwide and have an important ecological value, we are still far from understanding how these animals respond to pathogens. Mussels (*Mytilus galloprovincialis*) are present on all the continents, and they are even considered as a powerful invasive species^1,2^. Their ubiquitous presence and their sessile life explain why mussels have been selected and used as ecological markers for pollution for more than 40 years (mussel watch project)^3^. One of the most interesting characteristics of these invertebrates is the filtering activity that they have to use to feed themselves. On average, mussels can filter approximately 7.5 liters of water in one hour^4^, meaning they are in intimate contact with millions of microorganisms that are potentially pathogenic to them^5^. In fact, Stabili *et al*.^6^ reported that the abundance of *Vibrio spp*. is higher in mussels than in the surrounding water. Bivalves are susceptible to numerous diseases that compromise their culture, producing economic losses all over the world. As an example, the ostreid herpesvirus 1 (OsHV-1) has caused massive mortalities in oysters (*Crassostrea gigas*) in different parts of the world^7,8^. Mussels that cohabitate in the same areas as oysters and clams have experienced massive mortalities due to viral and bacterial infections. However, high mortalities have never been reported in the field for *M*. *galloprovincialis*^9,10^.

Mussels, as all invertebrates, have only an innate immune system to fight against pathogenic microorganisms. It is well known that invertebrates recognize conserved pathogen-associated molecular patterns (PAMPs)^11,12^, danger signals, and danger/damage-associated molecular patterns (DAMPs)^13,14^, yet it is not clear how bivalves, and mussels in particular, react to external stimuli taking into account their constant contact with microorganisms. To understand the immune reaction of these animals, a considerable effort has been made in recent years to increase the genomic and transcriptomic resources in bivalves and specifically in the Mediterranean mussel, *M*. *galloprovincialis*^15–20^.

To date, all the genomic studies carried out in mussels have been conducted using biological pooled samples and have not analyzed the response of individuals. However, we know that mussels have immune effectors that are tremendously variable within the population^21–23^. This is the case of myticins, which are antimicrobial peptides highly expressed in mussels^24^. There are three different myticin genes, named A, B and C, with similar DNA sequences and physicochemical properties. Of them, myticin C is the most variable AMP in mussels with the broader biological properties^25^.

In this work, we analyzed the transcriptomic response of six individual naïve mussels and how they respond to a simple injury (control animals injected with filtered sea water) or against a bacterial challenge (infected animals with *Vibrio splendidus*). Our results highlight the variability of the response in individual mussels and the importance of the appropriate experimental controls since the injection on the adductor muscle (the usual method to experimentally infect mussels) might be interpreted by the organism as a danger signal that may influence the unexpected regulation of several expressed genes.

## Methods

### Animals

Adult *M*. *galloprovincialis*, 8–10 cm in shell length, were obtained from a commercial shellfish farm (Vigo, Galicia, Spain) and maintained in open-circuit filtered seawater tanks at 15 °C with aeration. The animals were fed daily with *Phaeodactylum tricornutum* and *Isochrysis galbana*. Prior to the experiments, the animals were acclimatized to aquarium conditions for at least one week.

### Experimental design

**Figure 1:**
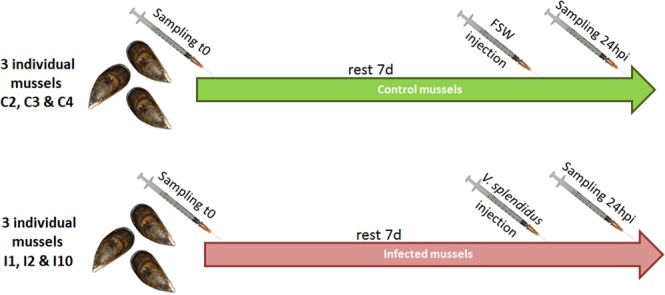
Diagram of the experimental design followed by the stimulation of individual mussels. Ten individual mussels were used per condition (control or infection), but only the three selected mussels for sequencing, in each condition, were represented in the experimental design.

A schematic representation of the experimental design is shown in Fig. 1. Twenty naïve mussels were marked and notched on the shell, and hemolymph (500 µl) was withdrawn from the adductor muscle of each mussel with a 0.5 mm diameter (25 G) disposable needle. This was the time zero (t0) sampling. The hemolymph was centrifuged at 4 °C at 3,000 g for 10 min, and the pellet was resuspended in 500 µl of TRIzol (Invitrogen), immediately homogenized with syringe and a 0.5-mm-diameter (25 G) disposable needle and stored at −80 °C until RNA isolation.

After one week, 10 of the mussels were injected in the adductor muscle with 100 µl of filtered seawater (FSW). The other 10 mussels were injected in the same way with 100 µl of a solution of *Vibrio splendidus* (reference strain, LGP32) at a non-lethal concentration (1 × 10^7^ CFU/mL). One day after the challenge, 24 hours post injection (24 hpi), hemolymph (500 µl) was sampled again from individual mussels and centrifuged in the same manner described above, and the pellet was resuspended in 500 µl of TRIzol. Samples were immediately homogenized with syringe and 25 G needle and kept at −80 °C until RNA isolation.

### RNA isolation, cDNA production and Illumina sequencing

RNA isolation was carried out in the 40 samples (n = 20 naïve at t0, n = 10 FSW injected at 24hpi, and n = 10 bacteria injected at 24hpi) using TRIzol and following the manufacturer’s protocol. Purification of RNA after DNase I treatment was performed with RNeasy mini (Qiagen). Next, the concentration and purity of the RNA was measured using a NanoDrop ND1000 spectrophotometer (NanoDrop Technologies, Inc.), and RNA integrity was tested on an Agilent 2100 Bioanalyzer (Agilent Technologies) before producing cDNA libraries for Illumina sequencing. Only the individuals with the best RNA samples (in terms of RNA quantity and quality) from both sampling points were chosen for Illumina sequencing: control n° 2 (C2), control n° 3 (C3) control n° 4 (C4), infected n° 1 (I1), infected n°2 (I2) and infected n° 10 (I10). In total, 12 RNA samples (2 per individual, the first at t0 and the second 24hpi of FSW or bacteria) were sequenced (details in Table 1).

**Table 1:** Summary of the transcriptome bioinformatics pipeline.

Reads origin	raw	trimmed
C2 t0	78,426,948	99.59%
C3 t0	44,346,854	98.03%
C4 t0	100,814,198	99.59%
I1t0	17,696,894	98.08%
I2 t0	93,114,098	99.60%
I10 t0	96,780,602	99.64%
C2 24 h	95,296,484	99.49%
C3 24 h	51,708,988	97.62%
C4 24 h	92,661,282	99.65%
I124 h	52,102,302	98.77%
I2 24 h	90,965,875	99.52%
I10 24h	99,262,970	99.55%
**Assembly**		
Contigs	270,324	
Range contig length	200–15,624	
Average contig length	512	
N50	574	
**Blast**		
Contigs identified by Uniprot/SwissProt	24.97%	
Contigs identified by molluscs database	54.93%	
**GO analysis**		
Annotated contigs	24.87%	
**KEGG analysis**		
Pathway assigned contigs	8.03%	

The mRNA-Seq sample preparation kit from Illumina was used according to the manufacturer’s instructions. mRNA was extracted from total RNA using oligo (dT) magnetic beads and cleaved into short fragments using fragmentation buffer. A cDNA library compatible with the Illumina NGS technology was then prepared from the fragmented mRNA via reverse transcription, second-strand synthesis and ligation of specific adapters (paired-ends) after cDNA purification using the QIAquick PCR Purification Kit (Qiagen). The amount of cDNA in each library was quantified through spectrofluorometric analysis using the Qbit system. Next-generation sequencing was performed using Illumina HiSeq™ 4000 technology at Macrogen Inc. Korea (Seoul, Republic of Korea).

### Bioinformatics and RNA-Seq

CLC Genomics Workbench, v.10.0.1 (CLC Bio; Qiagen) was used to filter, assemble and perform the RNA-Seq and the statistical analyses of individual mussels.

Raw reads were trimmed to remove low quality sequences (quality score limit 0.05 = PHRED 13), adaptor sequences, and sequences shorter than 70 bp. Then, a reference global transcriptome of the six mussels was assembled with an overlap criterion of 70% and a similarity of 90% to exclude paralogous sequence variants. The settings used were a mismatch cost = 2, deletion cost = 3, insert cost = 3, and minimum contig length = 200 base pairs.

Before the expression analysis, a subsampling step of the trimmed reads was carried out. A random subset of sequences was generated to equal the number of reads present in each sample. Then, RNA-Seq analysis of the subsamples (mismatches = 2, length fraction = 0.8, similarity fraction = 0.8, and maximum hits per read = 10) were performed. The expression values were set as transcripts per million (TPM). Finally, a differential expression analysis test (a Robinson and Smyth’s Exact Test, which assumes a Negative Binomial distribution of the data and takes into account the overdispersion caused by biological variability) was used to compare expression levels in each sample and to find the differentially expressed genes (DEGs). Transcripts with absolute fold change (FC) values >2 and Bonferroni corrected p-value < 0.05 were retained for further analyses.

### BLAST annotation, GO assignment, enrichment and KEGG analysis

Uniprot/Swissprot BLASTx results were used to obtain the Gene Ontology (GO) term assignments of the contig list with the Blast2GO software^26^. To improve the percentage of identification from the contig list, it was also annotated with an inhouse built database made with all of the mollusc sequences present in the NBCI nucleotide database. In both blast approaches, the e-value threshold was set at 1e-3. Then, the enrichment analyses of the up and down regulated DEGs (test set) were conducted, including the global mussel transcriptome as the reference set. A Fisher’s exact test was run with default values and a p-value cut-off of 0.05. The option to reduce the enriched list to the most specific GO terms was used. Only over-represented biological process (BP) terms were further analyzed. The Kyoto Encyclopedia of Genes and Genomes (KEGG) pathways in which the DEGs were involved were also analyzed with Blast2GO and summarized following the existent categories in the KEGG database (http://www.genome.jp/kegg/pathway.html).

### Flow cytometry analysis

**Figure 2:**
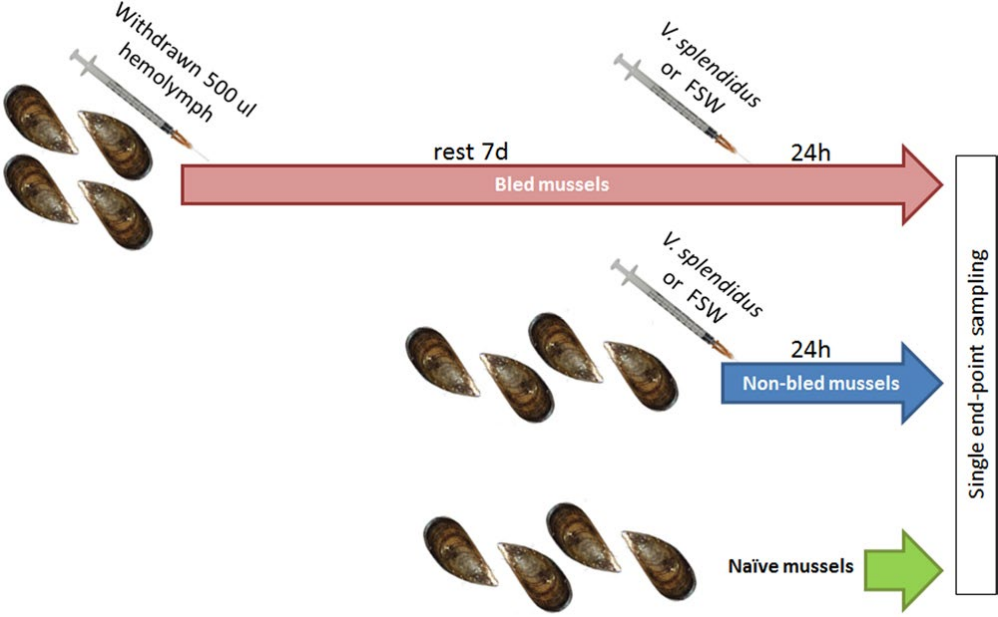
Experimental design for immunocytochemistry FACS detection of myticin C protein in mussel hemocytes. Four individual mussels were used for each experimental condition (naïve, non-bled FSW, non-bled Vibrio, bled FSW and bled Vibrio).

The distribution of myticin C in different cell populations was assayed in mussel hemocytes by immunocytochemistry and flow cytometry (FACSCalibur; BD). The experimental design is depicted in Fig. 2. Briefly, the studied conditions were five: (1) naïve mussels, (2) mussels injected with FSW, (3) mussels injected with *V*. *splendidus*, (4) mussels bled, let rest for 1 week and injected with FSW and (5) mussels bled, let rest for 1 week and injected with *V*. *splendidus*. A single end-point sampling was performed for each condition. Four mussels per condition were analyzed.

For each condition, 1.5 mL of hemolymph from individual mussels was withdrawn and immediately fixed in a final concentration of 2% paraformaldehyde (PFA). Samples were divided into three aliquots; the first sample was to analyze myticin C production through a custom antibody^25^, a control in which prebleed serum was used instead of the primary antibody and an absolute control without primary antibody. The immunocytochemistry protocol was performed as previously described^25^. The cell suspension was fixed for 15 min at 4 °C and washed twice for 10 min in phosphate buffered saline buffer (PBS) prior to permeabilization and staining with the anti-myticin C antibody (1:100). After overnight staining, samples were washed once for 10 min (PBS, 0.1% saponin, 0.2% bovine serum albumin, BSA) and incubated in the dark for 50 min at room temperature with secondary antibody (1:500). Finally, samples were washed for 10 min (PBS), and 200 µl of each condition and replicate was dispensed in a 96-well plate to be analyzed by fluorescence-activated cell sorting (FACS). The density plots and histograms were generated using Cell Quest Pro software (BD), and a one-way ANOVA with post-hoc analysis was used to analyze the significance of the results among all the conditions tested.

### Validation of RNA-Seq expression in response to tissue injury

A new experiment, including a new non-injected control, was carried out following the same layout as the RNA-Seq experimental design with new biological samples (Supplementary Fig. 1A). Briefly, individual mussels were sampled at t0, let them rest for one week and then divided in three tanks: (1) mussels injected with *V*. *splendidus* (1 × 10^7^ CFU/mL), (2) mussels injected with FSW and (3) mussels remained non- injected. One day after the challenge hemolymph was sampled again from individual mussels and RNA was extracted as previously described. cDNA was synthesized from each individual mussel with 200 ng of total RNA using NZY First-Strand cDNA Synthesis Kit (nzytech) following the manufacturer’s protocol.

Gene expression of selected genes (Supplementary Fig. 1B) was analyzed in a 7300 Real Time PCR System (Applied Biosystems). One microliter of fivefold-diluted cDNA template was mixed with 0.5 ml of each primer (10 mM) and 12.5 ml of SYBR Green PCR master mix (Applied Biosystems) in a final volume of 25 ml. The standard cycling conditions were 95 °C for 10 min, followed by 40 cycles of 95 °C for 15 s and 60 °C for 30 s. All reactions were performed as technical triplicates. The relative expression levels of the genes were normalized using 18 S as a reference gene following the Pfaffl method and standardized to the normalized expression of the t0 samplig to calculate fold changes. An independent t-test was used to analyze differences among conditions and differences were considered significant with p-value < 0.05. For the validation of the RNA-Seq vs the qPCR results linear regression and correlation were performed to analyze the studied genes and conditions.

## Results

### Assembly and annotation of mussel transcriptome

A summary of the sequence origin, assembly, identification, and annotation results is shown in Table 1. An average of 76 million raw reads was obtained from each individual sample of *M*. *galloprovincialis* hemocytes. The CLC Genomics Workbench was used to filter the raw reads, and over 97% of raw reads successfully passed the quality control in all of the samples. The assembly step was performed with all the samples available to obtain a global mussel transcriptome; 270,324 contigs were assembled with an average length of 512 bp. The putative identities of these sequences were obtained by Blast by two different means; Blast2GO software was used to identify the 24.97% of the contigs through a BLASTx approach against Uniprot, and CLC was used to identify 99.94% of the contigs using an inhouse designed database with all the sequences available in NCBI for molluscs. GO terms were assigned to 24.87% of the contigs and enzyme codes to find KEGG pathways to 8.03% of the sequences.

### Mussel transcriptome after bacterial or DAMP stimulation

**Figure 3:**
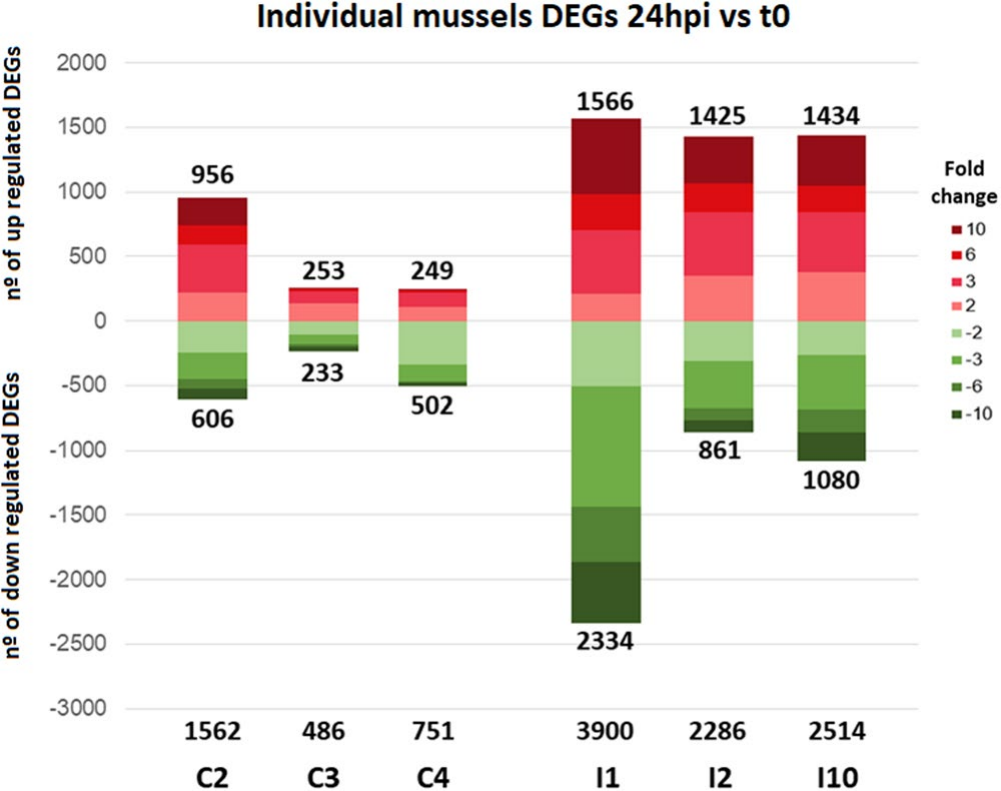
General overview of the expression values of mussels after bacterial challenge (infected) or FSW injection (controls). Stacked column chart reflecting the fold change distribution of DEGs in control and infected animals 24 hpi with regard to their own t0 sampling point.

The experimental design allowed us to sample hemolymph from each individual mussel before and after injection with bacteria or FSW; therefore, the real behavior of the modulated genes could be followed in each animal. Figure 3 shows the distribution of the differentially expressed genes (DEGs) in control and infected animals 24 hpi with regard to their own t0 sampling point. An expected response with more DEGs in infected animals (I1: 3,900 DEGs, I2: 2,286 DEGs, I10: 2,514 DEGs) compared to control animals (C2: 1,562 DEGs, C3: 486 DEGs, C4: 751 DEGs) was found. To validate the results of the RNA-Seq, the expression of IRG1, IFI44, MyD88, SOCS2, Myticin C, C1q and apextrin have been studied by qPCR. The statistical approach showed a significant correlation between the fold changes obtained with the two methodologies, validating our results (Supplementary Fig. 1C).

**Figure 4:**
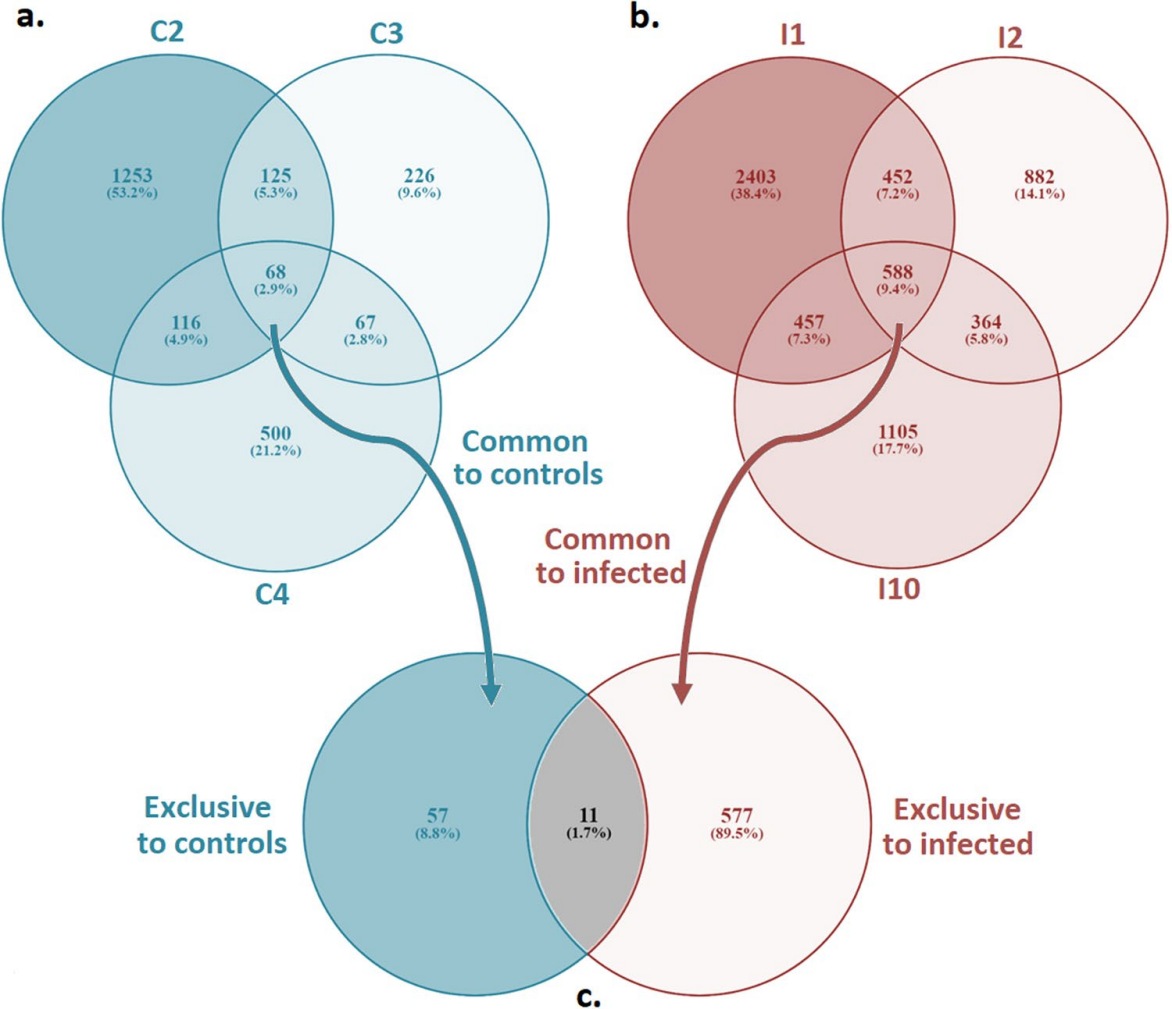
Venn diagrams of DEGs for each condition. (**a**) in blue, control mussels. (**b**) in red, infected mussels. (**c**) in gray, common genes to control and infected mussels.

We analyzed the effect of the injection in each animal and, surprisingly, a very small percentage of the modulated genes were shared between the 3 controls (only 2.9%) and the 3 infected animals (only 9.4%) as shown in the Venn diagrams in Fig. 4a,b. This result could mean that each mussel responds to the same stimulus in a different way. A simple injection affected the gene expression of control mussels in which a mean of 933 DEGs were significantly modulated after this danger signal. It has to be taken into account that a non-injected control after one-week rest is lacking in this experimental design for Illumina sequencing and RNA-Seq analysis. However, it was indeed included in a complementary experimental design and the gene expression of some immune-genes was analyzed by qPCR (Supplementary Fig. 1D). Results showed no significant differences between the initial and end-point sampling in non-injected controls except for C1q.

Of the total genes, 89.5% was modulated by the infection, and 8.8% was regulated after the tissue injury without bacterial challenge. When the common transcripts shared by the 3 controls and the 3 infected animals were compared (Fig. 4c), there were only 11 common contigs. Of them, five had informative annotations: C1q domain containing protein MgC1q61 (related to recognition in molluscs), chromobox protein homolog 7 (with epigenetic functions), putative gastrointestinal growth factor xP4 (important in mucosal protection and reconstitution), transcription factor AP-1 (a key transcription factor for immunity) and heat shock protein beta-1 (with numerous biological roles including regulation of stress resistance, inflammation and apoptosis). All these genes, regulated both in control and infected mussels, are directly or indirectly related to the defense response.

**Figure 5:**
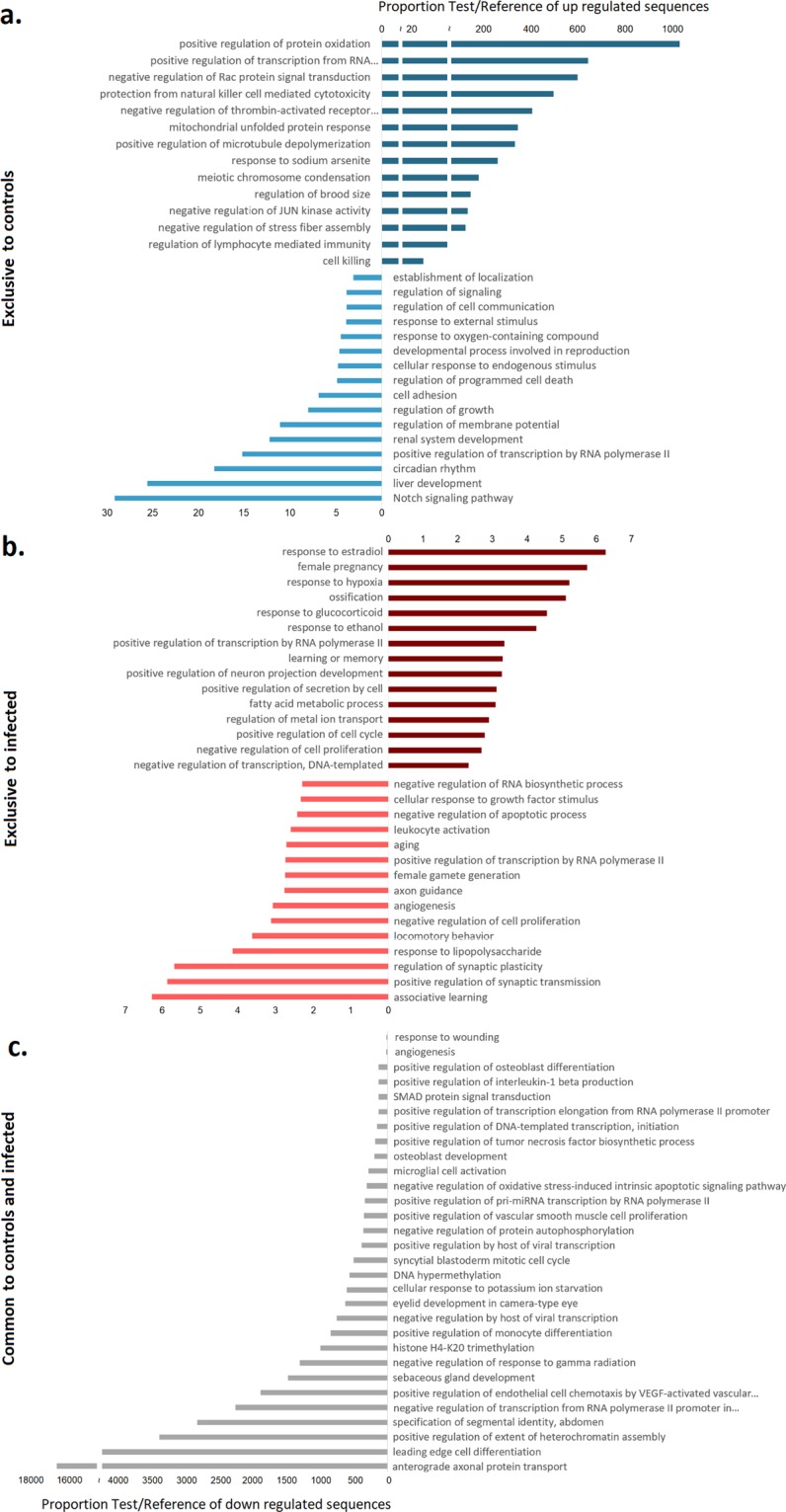
Enrichment analysis of DEGs. The proportions of test/reference sequences for up and down regulated genes are described following this legend: (**a)** blue, exclusive genes for control mussels; (**B**) red, exclusive genes for infected mussels; (**C**) gray, common genes to control and infected mussels.

The enrichment analysis of the GO terms in each compartment of the Venn diagram showed in Fig. 4c revealed 100 biological processes (BP) over-represented in the exclusive DEGs for controls, 439 for infected mussels and 37 in the common DEGs list for control and infected mussels. Figure 5 represents the top 30 most significant BP in these groups, divided into up or down regulated according to the fold change of the DEGs that they represent. The enrichment analysis revealed that the BP differentially represented in the common DEGs between control and infected mussels were related to immune terms such as “positive regulation of endothelial cell chemotaxis” representing the heat shock protein 27, “positive regulation of monocyte differentiation” or “negative regulation by host of viral transcription”, both terms related to the transcription factor AP-1. GO terms related to histone or DNA methylation were also present: “histone H4-K20 trimethylation” and “DNA hypermethylation”, representing the chromobox protein homolog 7. This result could indicate that both control and infected animals were subjected to a stimulus with similar capability to trigger epigenetic changes to respond to damage or infection. In the group of exclusive DEGs of control mussels, unexpected results were found, with BPs related to the immune response, antimicrobial peptides, and more specifically, to myticins, such as “cell killing”. Also in the control group, there were more processes related to the immune response both in the up and down regulated genes such as, in the up-regulated group, “regulation of lymphocyte mediated immunity”, “protection from natural killer cell mediated cytotoxicity” both terms related to serpin A3C, and, in the down-regulated group, “response to external stimulus”, “regulation of programmed cell death” terms representing genes such as the cell death specification protein 2, the GIMAP 4 or the superoxide dismutase. In the group of DEGs exclusive to infected mussels, the most interesting processes found were the ones linked to response to hypoxia, glucocorticoid, lipopolysaccharide and those related to cell proliferation, cell cycle or leucocyte activation, an indication of active hemocyte division and proliferation, representing 70 genes such as the suppressor of cytokine signaling 2, the ghrelin receptor, the fos-related antigen 2, the ficolin 2, the fibrinogen C domain-containing protein 1 and several growth and transcription factors.

**Figure 6:**
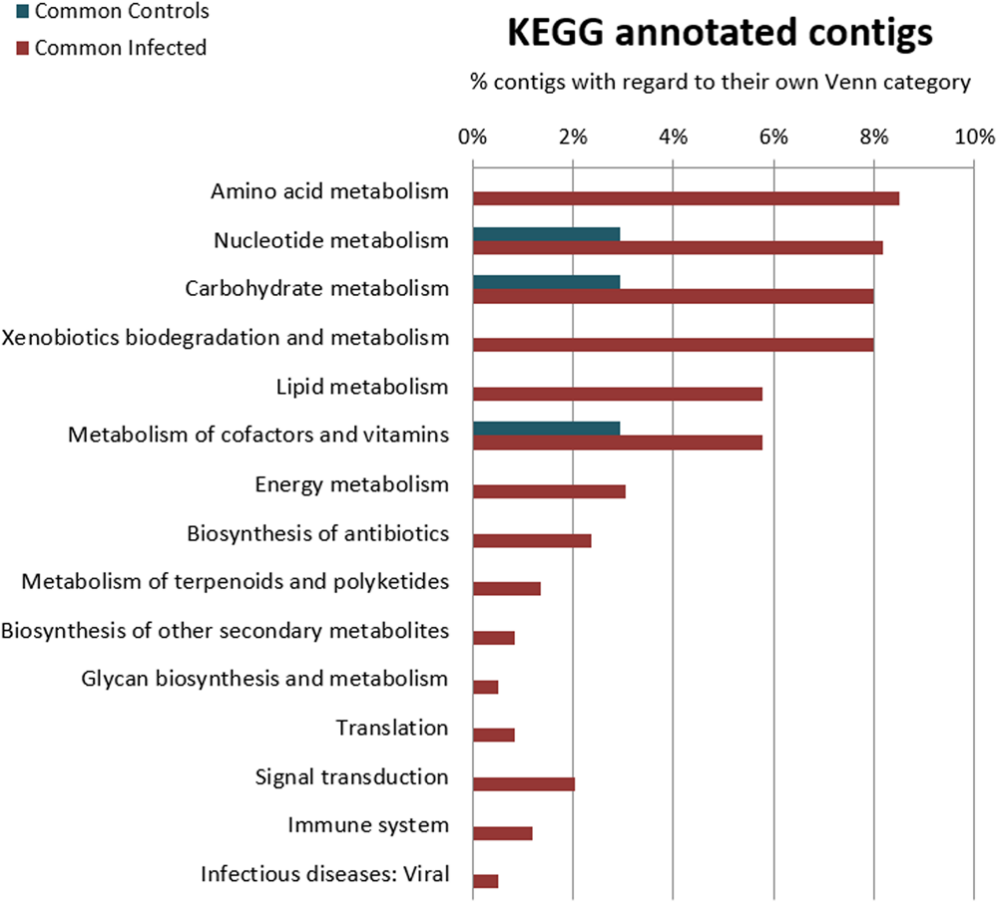
Summary of the KEGG reference pathway results for the significantly regulated contigs before and after injection: common for control mussels (blue) and common for infected mussels (red).

The KEGG reference pathway analysis showed, in the absolute number of DEGs, that infected mussels always had the maximum representation in every pathway. As we wanted to represent these results proportionally, we calculated the percentage of the DEGs ascribed to every pathway with regard to the modulated genes in each Venn category. This analysis is summarized in Fig. 6, which shows that metabolism was affected in different ways in control and infected animals. The pathways with more representation in control mussels were related to nucleotide and carbohydrate metabolism and to the metabolism of cofactors and vitamins. In infected mussels, the great majority of DEGs were included in metabolism pathways known to be greatly affected by immune challenges and in pathways related to translation, signal transduction (phosphatidylinositol signaling system, the mTOR signaling pathway and the PI3K-Akt signaling pathway), viral infectious diseases and immune system (defense cells differentiation and signaling) (Supplementary File 1). The PI3K, Akt and mTOR pathways are linked to the JAK-STAT signaling pathway, and their regulation is intimately related to the immune response and the regulation of processes such as cell proliferation, autophagy and apoptosis^27^, in line with the enrichment analysis results for infected mussels.

With the aim of knowing more about the nature of these DEGs, we focused on the most expressed genes in each group (complete data in Supplementary Table 1). Tables 2, 3 and 4 show, respectively, the top 25 genes found exclusively in controls, in infected animals and all those regulated genes shared between both of them, with the fold changes for each individual mussel. It is worth mentioning that antimicrobial peptides (AMPs) such as myticins or myticusin were highly up regulated in FSW injected animals (Table 2) and not in the infected animals, most likely reflecting a reaction against an injury or a danger signal without pathogens. Interestingly, in the DEGs found only in controls, genes related to cell proliferation, differentiation or cell activation were also up regulated and include elastin microfibril interface-located protein 2 (EMILIN-2), stathmin, low affinity epsilon Fc receptor, cell division cycle-associated protein 2 (CDCA2), and signal transducer and transcription activator (STAT). Some of these are associated with the cytoskeleton and cell motility (for example stathmin or the myticins that increase chemotaxis). The most downregulated genes in controls were related to the regulation of cell death: cell death specification protein 2, immediate early response gene 5 protein, protocadherin Fat 1, and GTPase IMAP family member 4.

**Table 2:** Top 25 up-regulated DEGs associated to tissue injury (controls). FC: fold change.

Contig ID	Description	FC C2	FC C3	FC C4	Mean	Mean p-val
Mg_109517	Myticin C	30.24	2.21	5.25	12.57	<0.0001
Mg_7618	Myticin B	25.00	2.09	2.85	9.98	<0.0001
Mg_127788	Myticin C	21.31	2.67	2.40	8.79	<0.0001
Mg_1042	Elastin microfibril interface-located protein 2	20.09	2.37	3.26	8.57	<0.0001
Mg_33725	Myticin C	15.24	2.99	2.08	6.77	<0.0001
Mg_5071	Stathmin	9.22	5.35	3.99	6.19	0.0003
Mg_7746	Myticusin-alpha precursor	6.54	7.31	4.20	6.02	<0.0001
Mg_150217	Myticusin-alpha precursor	6.40	6.71	4.22	5.78	<0.0001
Mg_37432	Heavy metal-binding protein HIP	8.39	3.62	4.20	5.40	<0.0001
Mg_26476	Low affinity immunoglobulin epsilon Fc receptor	7.49	3.56	2.71	4.59	<0.0001
Mg_27764	Cell division cycle-associated protein 2	6.83	4.22	2.56	4.54	0.0001
Mg_3246	Serine protease inhibitor A3C	7.60	3.79	2.15	4.51	<0.0001
Mg_5822	Signal transducer and transcription activator	3.89	2.45	2.98	3.11	0.0001
Mg_4540	Signal transducer and activator of transcription 2	4.12	2.43	2.33	2.96	0.0056
Mg_31595	Structural maintenance of chromosomes protein 4	3.06	2.89	2.82	2.93	0.0018
Mg_1195	Neuronal PAS domain-containing protein 4	−3.25	−3.09	−3.92	−3.42	<0.0001
Mg_1629	Sterol regulatory element-binding protein 1	−3.81	−5.01	−2.54	−3.79	<0.0001
Mg_23409	Perlucin	−5.22	−3.84	−5.89	−4.98	<0.0001
Mg_20788	GTPase IMAP family member 4	−6.73	−2.92	−6.03	−5.23	<0.0001
Mg_157047	Protocadherin Fat 1	−5.63	−5.08	−5.87	−5.52	<0.0001
Mg_29303	Notch homolog 2 N-terminal-like protein	−5.48	−5.01	−6.44	−5.64	<0.0001
Mg_6692	Immediate early response gene 5 protein	−2.45	−12.17	−10.66	−8.43	<0.0001
Mg_10957	Cell death specification protein 2	−3.92	−13.93	−8.16	−8.67	<0.0001
Mg_23430	Cornifelin homolog B	−15.91	−3.61	−34.40	−17.97	0.0002
Mg_247	Putative ariadne-like RING finger protein R811	−89.09	−3.30	−27.59	−39.99	<0.0001

**Table 3:** Top 25 up-regulated DEGs associated exclusively to a *Vibrio* infection. FC: fold change.

Contig ID	Description	FC I1	FC I2	FC I10	Mean	Mean p-val
Mg_80300	DNA-directed RNA polymerase II subunit RPB1-like	441.85	89.21	1085.67	538.91	<0.0001
Mg_106	Cis-aconitate decarboxylase	655.70	221.92	370.69	416.10	<0.0001
Mg_457	Solute carrier family 12 member 8	25.41	66.52	563.75	218.56	<0.0001
Mg_599	Neuropeptide Y receptor type 2-like	276.93	89.07	288.15	218.05	<0.0001
Mg_261923	Tropomyosin-1	26.44	69.72	153.20	83.12	<0.0001
Mg_3149	Neuropeptide Y receptor type 2-like	72.77	11.07	144.70	76.18	<0.0001
Mg_1646	PDZ and LIM domain protein Zasp	50.97	117.84	32.44	67.08	<0.0001
Mg_784	Furin-like protease kpc-1	62.94	32.09	92.91	62.65	<0.0001
Mg_24087	Suppressor of cytokine signaling 2	164.09	17.37	4.69	62.05	<0.0001
Mg_32754	Zinc finger protein 26	79.10	48.15	29.36	52.20	<0.0001
Mg_19011	Phenylalanine–tRNA ligase alpha subunit	42.23	47.93	58.54	49.57	<0.0001
Mg_2877	Growth hormone secretagogue receptor type 1	92.45	38.46	6.31	45.74	0.0003
Mg_9258	Suppressor of cytokine signaling 2	103.63	12.17	2.85	39.55	0.0078
Mg_7966	Phenylalanine–tRNA ligase alpha subunit B	43.11	28.66	43.79	38.52	<0.0001
Mg_6277	Interferon-induced protein 44-like	22.76	27.78	57.73	36.09	<0.0001
Mg_3834	Myeloid differentiation primary response protein MyD88	66.35	23.48	17.24	35.69	<0.0001
Mg_10058	Phenylalanine–tRNA ligase alpha subunit	37.23	23.16	46.01	35.46	<0.0001
Mg_6296	Cyclic GMP-AMP synthase	64.94	17.83	12.45	31.74	<0.0001
Mg_17428	Complement C1q-like protein 4	−40.12	−25.04	−40.69	−35.28	<0.0001
Mg_4119	Collagen alpha-1(XIV) chain	−53.14	−30.65	−28.95	−37.58	<0.0001
Mg_21146	Heat shock protein	−53.09	−47.87	−25.07	−42.01	<0.0001
Mg_44317	Nacre apextrin-like protein 1	−84.47	−8.82	−39.92	−44.40	0.0140
Mg_3338	Headcase protein	−156.82	−16.21	−19.39	−64.14	<0.0001
Mg_11571	Protein NDRG1	−59.97	−87.36	−75.87	−74.40	<0.0001
Mg_24372	Complement C1q tumor necrosis factor-related protein 4	−214.51	−18.16	−19.81	−84.16	<0.0001

**Table 4:** Regulated DEGs commons to controls and infected animals. FC: fold change.

Contig ID	Description	FC C2	FC C3	FC C4	Mean C	FC I1	FC I2	FC I10	Mean I	I vs C p-value	Sifnificance
Mg_81902	C1q domain containing protein MgC1q61	7.96	8.54	6.85	7.78	−2.37	−3.77	−2.47	−2.87	<0.0001	****
Mg_257	Putative gastrointestinal growth factor xP4	3.67	2.28	2.27	2.74	−7.42	−2.16	−5.26	−4.95	0.0085	**
Mg_384	Chromobox protein homolog 7	−2.34	−2.14	−3.09	−2.52	−2.84	−2.40	−3.48	−2.91	0.4196	ns
Mg_234	Transcription factor AP-1	−5.12	−4.36	−3.55	−4.34	−15.56	−8.14	−7.55	−10.42	0.0811	ns
Mg_496	Heat shock protein beta-1	−290.15	−25.12	−52.75	−122.67	−82.31	−94.11	−80.22	−85.55	0.6821	ns

P-value and significance show the results of an independent t-test between the controls and the infected mussels.

In infected animals (Table 3), some of the exclusive DEGs were directly involved in the immune response, in concordance with the results of the KEGG pathways analysis (Fig. 6). Some of these genes that were always up regulated were IRG1, SOCS2, IFI44 and Myd88. The cis-aconitate decarboxylase (also known as Immune-responsive gene 1 protein or IRG1) is an enzyme of de tricarboxylic acid cycle which produces itaconate after an immune challenge. Itaconate has been recently studied for its anti-inflammatory and antimicrobial properties^28,29^, making a direct link between basic metabolism and the immune response. The suppressor of cytokine signaling 2 (SOCS2) is a well-known negative regulator of the JAK-STAT signaling cascade, whose function is to control the inflammatory response^30^. The myeloid differentiation primary response protein Myd88, an essential mediator of the Toll signaling pathway, has been characterized in bivalves, and it is up regulated after bacterial infections^31^, consistent with our results. The interferon-induced protein 44 (IFI44), on the other hand, has been less well studied, but its upregulation has been associated with viral infections also in bivalves^32–34^, and it is known to have antiviral properties^35^. However, its exact function is still unknown, and it could be possible that in bivalves, it also has antibacterial roles, such as its strong upregulation 24 hours after *V*. *splendidus* injection suggested. The presence of IFI44 in the infected DEGs list is a possible explanation for the “viral infectious diseases” category in the KEGG analysis.

Table 4 shows the regulated transcripts shared between controls and infected animals. Two of them (C1q domain containing protein MgC1q61 and Putative gastrointestinal growth factor xP4) were up regulated in all the controls but down regulated in infected mussels, and their gene expression was significantly different between control and infected mussels. Individual responses can override experimental design in many genes, but in this case, even despite individual variability, it was possible to detect experimentally induced changes in hemocyte gene expression. In the specific case of the C1q domain containing protein, it could be due to its role as a pathogen recognition protein and its fast upregulation after an infection, with a return to physiological levels within 24 h^23^. The putative gastrointestinal growth factor xP4 is a member of the trefoil factor family (TFF), a group of molecules with a pivotal role in maintaining the surface integrity of mucous epithelia *in vivo*, explaining its up-regulation after the injury in control mussels; although the exact role of the TFF peptides is unknown they are known to have antiapoptotic effects and probably modulate inflammatory processes^36,37^ a possible reason of its diverse behavior in control and infected samples. The other 3 annotated genes, always down regulated, were heat shock protein beta-1, associated with the acute phase response and found modulated after a tissue injury in *M*. *galloprovincialis* at the protein level^38^; chromobox protein homolog 7, related to epigenetics via chromatin remodeling and modification of histones^39^; and the transcription factor AP-1, known to control the expression of genes related to differentiation, proliferation and apoptosis, and intimately linked to NF-kB^40^.

### Expression of myticins

**Figure 7:**
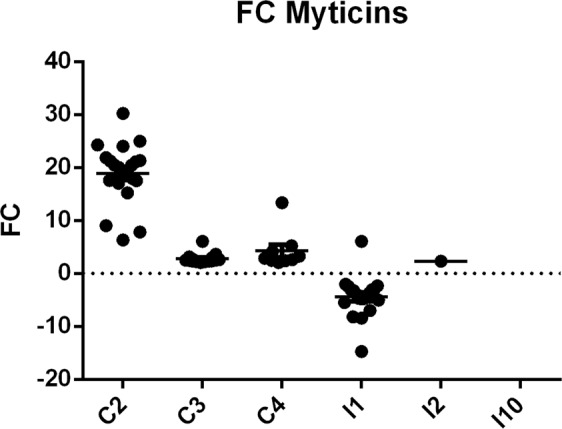
Expression profiles of myticins in control and infected individual mussels. Dots indicate the fold change (FC) of myticin transcripts 24 hpi with regard to their own t0. The line indicates the mean of the expression for each individual. Note the increase in the expression level in controls with regard to infected mussels and the great individual variability in the number of regulated transcripts, especially among infected mussels, with I10 showing no differentially expressed myticins.

The presence of myticins in the group of the most significantly up regulated genes after FSW injection led us to further investigate this fact. Taking into account that we had previously found that these molecules had antibacterial and antiviral properties^25,41,42^, we expected to find them being up regulated after bacterial challenge and not after a tissue injury, as they were in the controls. Figure 7 shows the expression of all the differentially expressed myticins in each individual. Control animals, after the injection of FSW, presented higher expression values than those challenged with *V*. *splendidus*, where a down regulation of myticins could be observed for only one (I2) or none (I10) of the myticin transcripts that were differentially expressed.

**Figure 8:**
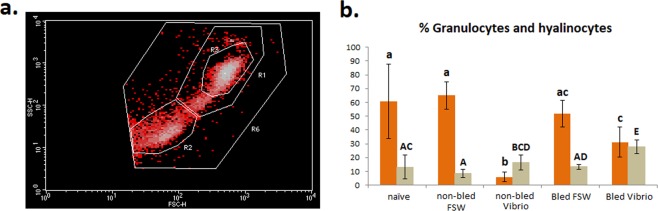
Description of hemocyte populations by flow cytometry. (**a**) Dot plot of the representative cell populations in mussel: R1, granulocytes; R2, hyalinocytes. Represented dots are gated for R6 region. (**b**) Description of the variation in hemocyte populations among samples: Orange, granulocytes; brown, hyalinocytes. Standard deviation is indicated for the four replicates. Different letters indicate significant differences (p-value < 0.05) among groups: low-case letters for granulocytes and capital letters for hyalinocytes.

To further confirm these unexpected results, we designed a new experimental protocol to determine if myticins had the same behavior at the protein level using flow cytometry. The experiment had the same layout as the one used to study the transcriptome but with more experimental controls: we included naïve and non-bled mussels to confirm the involvement of tissue injury in the expression of myticins (Fig. 2). Figure 8a shows the general profile of the hemocyte population, with R1 being the granulocytes and R2 the hyalinocytes. After *V*. *splendidus* infection in bled (injured) and non-bled mussels, there was a change in the hemocyte population structure with a significant reduction in the percentage of granulocytes with regard to their controls (FSW) and a significant increase in the percentage of hyalinocytes. These changes were present in bled and non-bled animals after infection, but the decrease in the granulocytes was less important in bled or injured mussels (Fig. 8b and Supplementary Table 3). It is noteworthy that the hemocyte population changed drastically after bacterial injection, but it recovered significantly, with regard to the non-bled mussels, if a previous damage stimulus had been made (Fig. 8b and Supplementary Fig. 2).

**Figure 9:**
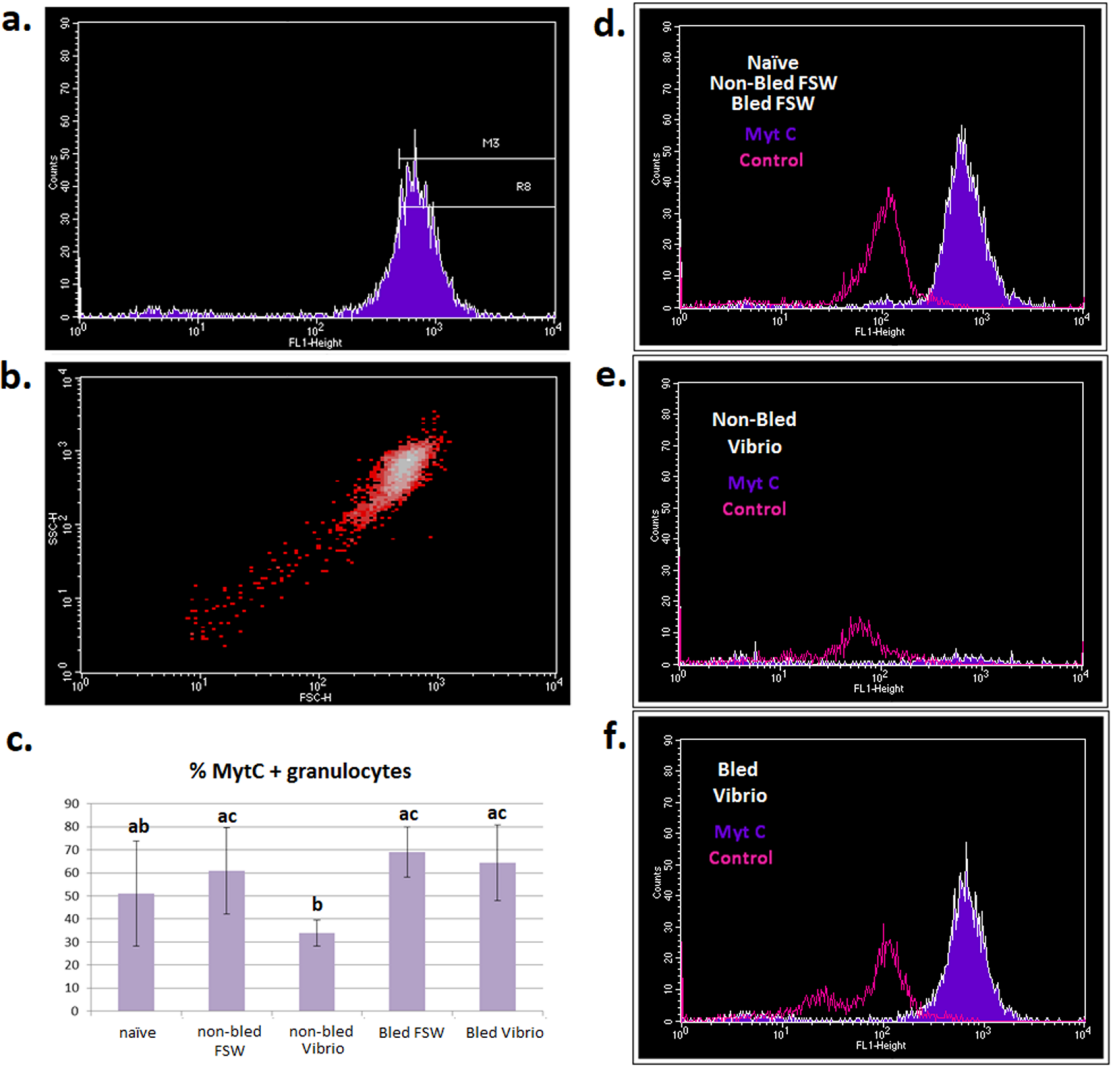
FACS results of the immunocytochemistry of myticin C. (**a**) Criteria to choose myticin C positive cells (M3). (**b**) Myticin C positive cells in a representative sample. (**c**) Statistical analysis of the percentage of granulocytes positive for myticin C immunocytochemistry. Standard deviation is indicated for the four replicates. Significant differences (p-value < 0.05) among groups are indicated by different letters. (**d**–**f**) Representative overlay of histograms for FL1 intensity of granulocytes (R1) population.

In Fig. 9a, the region considered positive for myticin C expression is shown. These positive cells corresponded to the granulocyte (R1) population (Fig. 9b). When the production of myticin C was evaluated after the different treatments, a significant decrease in the population positive for myticin C was found after *Vibrio* infection (Fig. 9c and Supplementary Table 3). However, this reduction was not observed when mussels were bled before, generating a tissue injury. The percentage of myticin C positive granulocytes was also significantly higher in mussels previously bled and then infected with bacteria when compared with mussels only injected with bacteria. Figure 9d-f illustrate representative R1 FL1 profiles for every sample group. Naïve or control mussels injected with FSW showed a granulocyte population positive for myticin C (Fig. 9d) that was lost in *Vibrio* challenged mussels without a previous stimulation (Fig. 9e). However, the bled/injured mussels after a *Vibrio* challenge did not lose the myticin C labeling (Fig. 9f) probably because of the effect of the previous stimulus that prevented the hemocyte population from decaying after a subsequent bacterial infection.

## Discussion

Next generation sequencing technologies are particularly valuable in the study of non-model organisms because they supply the scarce availability of reference genomes, and lead the way to understand many biological processes that could not be studied due to the lack of cellular lines or antibodies. Most of the transcriptomic analyses in the field of bivalves have been conducted using pools of animals or tissues^43,44^. When biological materials, such as hemocytes, are scarce, this is a good solution^45–48^. Biological pools help us to eliminate individual differences and concentrate on the clear patterns of a particular experiment; determining, for example, how bivalves react to a bacterial infection^45–48^. Although this approach is totally correct, we cannot forget that we are dealing with wild animals. They are not laboratory homogeneous strains, and they differ in their genetic and physiological backgrounds. In this work, we were interested in the specific transcriptomic response of individual mussels before and after a bacterial infection.

The first unexpected result occurred when we realized that the percentage of DEGs shared among 3 different mussels after the same stimulus was very small. Each individual had its own repertoire of genes, and this result occurred both in controls (reaction of 3 individuals against FSW) and in infected mussels (reaction of 3 individuals against *V*. *splendidus*). The high diversity found in mussels for many genes such as antimicrobial peptides (AMPs), fibrinogen-related proteins (FREPs) or C1q domain containing proteins^21–23^ increased the differences among individual mussels, which express a specific repertoire of transcripts. The interindividual variability in the basal expression of AMPs was previously reported in oysters and mussels^15,21,49^. The variability of bivalve responses would be related to the antigenic environment of each individual and would also show the genetic diversity of these animals. We wonder if we are losing important information when we analyze the immune response in pools of animals instead of using individuals and whether we are underestimating the possibility of the individual immunity as part of a possible social immunity of mussels^50^, as their impressive resilience and survival capacities point out.

The second striking result was that the 3 control mussels showed 1,562, 486 and 751 DEGs (using this statistical threshold: FC > |2| and Bonferroni corrected p-value < 0.05), which were probably modulated by a simple tissue injury (FSW injection). The hemocytes are the bivalve immunocompetent cells, but they are also involved in other physiological processes such as basic homeostasis and wound and shell repair^51,52^. As mussels host diverse and abundant microbial communities, they probably do not respond to all the microorganisms that they are in contact with. Only if there is tissue damage can they trigger an immune reaction. Cell proliferation and migration related genes were overexpressed in control mussels: stathmin, key protein in the cell cycle regulation^53^; low affinity epsilon Fc receptor, with essential roles in defense cell growth and differentiation^54^; cell division cycle-associated protein 2, which controls the cell cycle^55^; signal transducer and transcription activator, member of the STAT family and regulator of cellular immunity, proliferation and differentiation^56^; and structural maintenance of chromosomes protein 4, a protein required to enter into mitotic phase^57^. In contrast, genes related to the regulation of cell death were down modulated: cell death specification protein 2, with a direct link to the activation of apoptosis^58^; protocadherin Fat 1, a tumor suppressor and inhibitor of cell migration^59^; and GTPase IMAP family member 4, member of the GIMAP family which may play a role in defense cells differentiation and apoptosis^60,61^. These results were in agreement with the flow cytometry results. When an initial injury or bleeding occurred, changes in hemocyte structure were milder compared with those observed after a bacterial infection. The response of bivalves to tissue injury or different danger signals has not been explored and deserves to be further investigated. These results also highlight the importance of using appropriate controls in our studies. In fact, the acclimation and rest period is important to rule out possible responses due to stress or handling^62^. The usual procedure of injection in the adductor muscle could induce an injury that mussels feel as a danger signal. Therefore, if our aim is to analyze the bivalve immune response against a pathogen, we must clearly identify this specific response without the reaction against the tissue damage produced by the injection. Although our transcriptome study did not take into account a non-injected control, in an additional experiment we could confirm that the initial hemolymph extraction did not induce significant changes in gene expression after one week except for C1q, a pleiotropic gene involved in innate immunity and also in shell repair^63^, a process triggered by the notch in the shell for hemolymph sampling.

The third finding emerged when we detected AMPs included in the top 25 most expressed genes from controls (exclusive to controls and not found in infected mussels) (Table 2). To date, the main function recognized for AMPs has been the direct killing of microorganisms^64^. We previously reported the biological characteristics of mussel myticins: activity against bacteria^41^, activity against molluscan, fish and human viruses^25,38^, chemotactic activity^25^, etc. Myticins are very diverse, but they are not usually regulated at the transcript level after infection, they are stored in hemocyte granules ready to act when needed^42^. However, even with these premises, it was unexpected that myticins were up regulated in controls. Their expression was increased after a tissue injury, a danger signal. A non-injected control after the rest period in the RNA-Seq could further confirm this results, and although it was not included in the initial experimental design, the qPCR analysis supports our observations that the response to a wound and to a pathogen is quite diverse. The same response is observed with myticusin in that although it has only been described as an antimicrobial peptide^65^, it probably can also be involved in cell proliferation and chemotaxis such as myticins are. Therefore, why a tissue injury and not a bacterial infection triggers their transcription? Our hypothesis is that a bacterial signal (PAMP) triggers the release of granules to let the AMPs fight the bacteria, and the tissue injury (DAMP) triggers the mechanisms to produce and store these AMPs. The explanation could be a new and unrecognized function of these AMPs due to exposure to many microorganisms, where wounds matter more than a potential pathogen.

In wild conditions a tissue damage is a possible cause of infection and it makes sense that these animals could be preparing themselves for a possible infection after being wounded. Interestingly, flow cytometry analysis suggests that the myticins could also help in the control of the infection process since the reaction triggered by the bleeding (tissue injury) was enough to avoid the granulocyte and the myticin C+ cell population decrease caused by bacteria, in concordance with the transcriptomic results, which show genes related to cell proliferation. Although further studies will be needed to confirm cell proliferation and hematopoiesis after tissue injury in *M*. *galloprovincialis*, these results open the door for new research topics regarding innate immunity in bivalves.

## Conclusions

In summary, we have raised the question of using an individual approach when facing mussel transcriptomics, which may be true for other organisms as well. We have demonstrated that every single individual expression profile can be very different in terms of the number of genes expressed and in the magnitude of their expression. A careful experimental design should be carried out especially with non-model species, as their individual transcriptomes can be quite diverse. Additionally, some common experimental procedures in bivalves such as shell notching or injection of treatments could trigger unexpected responses such as an unspecific inflammatory response or defense cell proliferation. We found that myticin C may play new roles in preparing the mussels for future pathogenic processes after a danger signal. A tissue injury is a breach of the first defense barrier, which could be easily followed by a subsequent infection.

More studies should be conducted in the future to understand more about these processes. However, currently, we undoubtedly can say that mussels are anything but simple and that more revelations will appear in the study of “non-model mussel immunity”.

## Supplementary information

Supplementary Information

## Data Availability

The raw reads of each sampling point and treatment were deposited in the NCBI database with the accession numbers SAMN09096971 to SAMN09096982. Accessible through https://www.ncbi.nlm.nih.gov/biosample/9096971 to…/909682.

## References

[1] Saarman NP, Pogson GH (2015). Introgression between invasive and native blue mussels (genus *Mytilus*) in the central California hybrid zone. Mol. Ecol..

[2] Lathlean JA (2016). Cheating the Locals: Invasive mussels steal and benefit from the cooling effect of indigenous mussels. PLoS One.

[3] Farrington JW (2016). Goldberg’s proposal of “the Mussel Watch”: Reflections after 40years. Mar. Pollut. Bull..

[4] Cole BE, Thompson JK, Cloern JE (1992). Measurement of filtration rates by infaunal bivalves in a recirculating flume. Mar. Biol..

[5] Suttle CA (2007). Marine viruses – major players in the global ecosystem. Nat. Rev. Microbiol..

[6] Stabili L, Acquaviva MI, Cavallo RA (2005). *Mytilus galloprovincialis* filter feeding on the bacterial community in a Mediterranean coastal area (Northern Ionian Sea, Italy). Water Res..

[7] Segarra A (2010). Detection and description of a particular Ostreid herpesvirus 1 genotype associated with massive mortality outbreaks of Pacific oysters, *Crassostrea gigas*, in France in 2008. Virus Res..

[8] Garcia C (2011). Ostreid herpesvirus 1 detection and relationship with *Crassostrea gigas* spat mortality in France between 1998 and 2006. Vet. Res..

[9] Romero A (2014). Occurrence, seasonality and infectivity of Vibrio strains in natural populations of mussels *Mytilus galloprovincialis*. Dis. Aquat. Organ..

[10] Domeneghetti S (2014). Mortality occurrence and pathogen detection in *Crassostrea gigas* and *Mytilus galloprovincialis* close-growing in shallow waters (Goro lagoon, Italy). Fish Shellfish Immunol..

[11] Medzhitov R, Janeway CA (1997). Innate immunity: the virtues of a nonclonal system of recognition. Cell.

[12] Khalturin K, Bosch TC (2007). Self/nonself discrimination at the basis of chordate evolution: limits on molecular conservation. Curr. Opin. Immunol..

[13] El Chamy L, Leclerc V, Caldelari I, Reichhart JM (2008). Sensing of ‘danger signals’ and pathogen-associated molecular patterns defines binary signaling pathways ‘upstream’ of Toll. Nat. Immunol..

[14] Berisha A, Mukherjee K, Vilcinskas A, Spengler B, Römpp A (2013). High-resolution mass spectrometry driven discovery of peptidic danger signals in insect immunity. PLoS One.

[15] Rosani U (2011). Massively parallel amplicon sequencing reveals isotype-specific variability of antimicrobial peptide transcripts in *Mytilus galloprovincialis*. PLoS One.

[16] Nguyen TT, Hayes BJ, Guthridge K, Ab Rahim ES, Ingram BA (2011). Use of a microsatellite-based pedigree in estimation of heritabilities for economic traits in Australian blue mussel. Mytilus galloprovincialis. J. Anim. Breed. Genet..

[17] Gerdol M (2014). RNA sequencing and de novo assembly of the digestive gland transcriptome in *Mytilus galloprovincialis* fed with toxinogenic and non-toxic strains of *Alexandrium minutum*. BMC Res. Notes.

[18] Moreira R (2015). RNA-Seq in *Mytilus galloprovincialis*: comparative transcriptomics and expression profiles among different tissues. BMC Genomics.

[19] Murgarella M (2016). A First Insight into the Genome of the Filter-Feeder Mussel *Mytilus galloprovincialis*. PLoS One.

[20] Moreira R (2018). Bivalve transcriptomics reveal pathogen sequences and a powerful immune response of the Mediterranean mussel (*Mytilus galloprovincialis*). Mar. Biol..

[21] Costa MM (2009). Evidence of high individual diversity on myticin C in mussel (*Mytilus galloprovincialis*). Dev. Comp. Immunol..

[22] Romero A (2011). Individual sequence variability and functional activities of fibrinogen-related proteins (FREPs) in the Mediterranean mussel (*Mytilus galloprovincialis*) suggest ancient and complex immune recognition models in invertebrates. Dev. Comp. Immunol..

[23] Gerdol M (2011). The C1q domain containing proteins of the Mediterranean mussel *Mytilus galloprovincialis*: a widespread and diverse family of immune-related molecules. Dev. Comp. Immunol..

[24] Pallavicini A (2008). High sequence variability of myticin transcripts in hemocytes of immune-stimulated mussels suggests ancient host-pathogen interactions. Dev. Comp. Immunol..

[25] Balseiro P (2011). *Mytilus galloprovincialis* myticin C: a chemotactic molecule with antiviral activity and immunoregulatory properties. PLoS One.

[26] Conesa A (2005). Blast2GO, a universal tool for annotation, visualization and analysis in functional genomics research. Bioinformatics.

[27] Xia P, Xu XY (2015). PI3K/Akt/mTOR signaling pathway in cancer stem cells: from basic research to clinical application. Am. J. Cancer Res..

[28] Lampropoulou V (2016). Itaconate links inhibition of succinate dehydrogenase with macrophage metabolic remodeling and regulation of inflammation. Cell Metab..

[29] Lua NHH, Medzhitov R (2016). Food Fight: Role of Itaconate and Other Metabolites in Antimicrobial Defense. Cell Metab..

[30] Rico-Bautista E, Flores-Morales A, Fernández-Pérez L (2006). Suppressor of cytokine signaling (SOCS) 2, a protein with multiple functions. Cytokine Growth Factor Rev..

[31] Toubiana M (2013). Toll-like receptors and MyD88 adaptors in Mytilus: complete cds and gene expression levels. Dev. Comp. Immunol..

[32] Pauletto M (2017). Long dsRNAs promote an anti-viral response in Pacific oyster hampering ostreid herpesvirus 1 replication. J. Exp. Biol..

[33] He Y (2015). Transcriptome analysis reveals strong and complex antiviral response in a mollusc. Fish Shellfish Immunol..

[34] Rosani U (2015). Dual analysis of host and pathogen transcriptomes in ostreid herpesvirus 1-positive Crassostrea gigas. Environ. Microbiol..

[35] Power D (2015). IFI44 suppresses HIV-1 LTR promoter activity and facilitates its latency. Virology.

[36] Hoffmann W, Jagla W (2002). Cell type specific expression of secretory TFF peptides: colocalization with mucins and synthesis in the brain. Int. Rev. Cytol..

[37] Conlon JM (2015). Host-defense and trefoil factor family peptides in skin secretions of the Mawa clawed frog Xenopus boumbaensis (Pipidae). Peptides.

[38] Franco-Martínez L (2018). Alterations in haemolymph proteome of *Mytilus galloprovincialis* mussel after an induced injury. Fish Shellfish Immunol..

[39] Li Q (2010). Polycomb CBX7 directly controls trimethylation of histone H3 at lysine 9 at the p16 locus. PLoS One.

[40] Fujioka S (2004). NF-kappaB and AP-1 connection: mechanism of NF-kappaB-dependent regulation of AP-1 activity. Mol. Cell. Biol..

[41] Martinez-Lopez A (2013). pH-dependent solution structure and activity of a reduced form of the host-defense peptide myticin C (Myt C) from the mussel *Mytilus galloprovincialis*. Mar. Drugs.

[42] Novoa B (2016). Antiviral activity of myticin C peptide from mussel: an ancient defense against herpesviruses. J. Virol..

[43] Zhao X (2017). Comparative transcriptome analysis of *Sinonovacula constricta* in gills and hepatopancreas in response to *Vibrio parahaemolyticus* infection. Fish Shellfish Immunol..

[44] Ren Y, Xue J, Yang H, Pan B, Bu W (2017). Transcriptome analysis of *Ruditapes philippinarum* hepatopancreas provides insights into immune signaling pathways under *Vibrio anguillarum* infection. Fish Shellfish Immunol..

[45] Moreira R (2012). Gene expression analysis of clams *Ruditapes philippinarum* and *Ruditapes decussatus* following bacterial infection yields molecular insights into pathogen resistance and immunity. Dev. Comp. Immunol..

[46] Tanguy M (2013). Sequence analysis of a normalized cDNA library of *Mytilus edulis* hemocytes exposed to *Vibrio splendidus* LGP32 strain. Results Immunol..

[47] Pauletto M (2014). Deep transcriptome sequencing of *Pecten maximus* hemocytes: a genomic resource for bivalve immunology. Fish Shellfish Immunol..

[48] Dong W, Chen Y, Lu W, Wu B, Qi P (2017). Transcriptome analysis of *Mytilus coruscus* hemocytes in response to *Vibrio alginnolyficus* infection. Fish Shellfish Immunol..

[49] Rosa RD, Alonso P, Santini A, Vergnes A, Bachère E (2015). High polymorphism in big defensin gene expression reveals presence-absence gene variability (PAV) in the oyster *Crassostrea gigas*. Dev. Comp. Immunol..

[50] Meunier J (2015). Social immunity and the evolution of group living in insects. Philos. Trans. R. Soc. Lond. B Biol. Sci..

[51] Fisher, W.S. Structure and functions of oyster hemocytes in *Immunity in Invertebrates* (ed. Brehélin, M.) Proceedings in Life Sciences (Springer, 1986).

[52] Bachère E (2015). The new insights into the oyster antimicrobial defense: Cellular, molecular and genetic view. Fish Shellfish Immunol..

[53] Rubin CI, Atweh GF (2004). The role of stathmin in the regulation of the cell cycle. J. Cell. Biochem..

[54] Acharya M (2010). CD23/FcεRII: molecular multi-tasking. Clin. Exp. Immunol..

[55] Uchida F (2013). Overexpression of CDCA2 in human squamous cell carcinoma: correlation with prevention of G1 phase arrest and apoptosis. PLoS One.

[56] Benekli M, Baer MR, Baumann H, Wetzler M (2003). Signal transducer and activator of transcription proteins in leukemia. Blood.

[57] Kimura K, Cuvier O, Hirano T (2001). Chromosome condensation by a human condensin complex in Xenopus egg extracts. J. Biol. Chem..

[58] Conradt B, Wu YC, Xue D (2016). Programmed cell death during *Caenorhabditis elegans* development. Genetics.

[59] Hu X (2018). FAT1 inhibits cell migration and invasion by affecting cellular mechanical properties in esophageal squamous cell carcinoma. Oncol. Rep..

[60] Carter C (2007). A natural hypomorphic variant of the apoptosis regulator Gimap4/IAN1. J. Immunol..

[61] Heinonen MT, Kanduri K, Lähdesmäki HJ, Lahesmaa R, Henttinen TA (2015). Tubulin- and actin-associating GIMAP4 is required for IFN-γ secretion during Th cell differentiation. Immunol. Cell. Biol..

[62] Thompson EL (2012). Optimal acclimation periods for oysters in laboratory-based experiments. J. Molluscan Stud..

[63] Arivalagan J (2017). Insights from the Shell Proteome: Biomineralization to Adaptation. Mol. Biol. Evol..

[64] Mitta G (2000). Differential distribution and defence involvement of antimicrobial peptides in mussel. J. Cel.l Sci..

[65] Liao Z (2013). Molecular characterization of a novel antimicrobial peptide from *Mytilus coruscus*. Fish Shellfish Immunol..

